# eHealth Program to Reduce Hospitalizations Due to Acute Exacerbation of Chronic Obstructive Pulmonary Disease: Retrospective Study

**DOI:** 10.2196/24726

**Published:** 2021-03-18

**Authors:** Amanda R van Buul, Caroline Derksen, Ouke Hoedemaker, Oscar van Dijk, Niels H Chavannes, Marise J Kasteleyn

**Affiliations:** 1 Department of Pulmonology Leiden University Medical Center Leiden Netherlands; 2 Bravis Department of Pulmonology Roosendaal Netherlands; 3 Medicine Men Utrecht Netherlands; 4 Department of Public Health and Primary Care Leiden University Medical Center Leiden Netherlands; 5 National eHealth Living Lab Leiden Netherlands

**Keywords:** COPD, eHealth, exacerbations, hospitalizations, mHealth

## Abstract

**Background:**

Hospitalization for acute exacerbation of chronic obstructive pulmonary disease (COPD) is associated with poor prognosis. eHealth interventions might improve outcomes and decrease costs.

**Objective:**

This study aimed to evaluate the effect of an eHealth program on COPD hospitalizations and exacerbations.

**Methods:**

This was a real-world study conducted from April 2018 to December 2019 in the Bravis Hospital, the Netherlands. An eHealth program (EmmaCOPD) was offered to COPD patients at risk of exacerbations. EmmaCOPD consisted of an app that used questionnaires (to monitor symptoms) and a step counter (to monitor the number of steps) to detect exacerbations. Patients and their buddies received feedback when their symptoms worsened or the number of steps declined. Generalized estimating equations were used to compare the number of days admitted to the hospital and the total number of exacerbations 12 months before and (max) 18 months after the start of EmmaCOPD. We additionally adjusted for the potential confounders of age, sex, COPD severity, and inhaled corticosteroid use.

**Results:**

The 29 included patients had a mean forced expiratory volume in 1 second of 45.5 (SD 17.7) %predicted. In the year before the intervention, the median total number of exacerbations was 2.0 (IQR 2.0-3.0). The median number of hospitalized days was 8.0 days (IQR 6.0-16.5 days). Afterwards, there was a median 1.0 (IQR 0.0-2.0) exacerbation and 2.0 days (IQR 0.0-4.0 days) of hospitalization. After initiation of EmmaCOPD, both the number of hospitalized days and total number of exacerbations decreased significantly (incidence rate ratio 0.209, 95% CI 0.116-0.382; incidence rate ratio 0.310, 95% CI 0.219-0.438). Adjustment for confounders did not affect the results.

**Conclusions:**

The eHealth program seems to reduce the number of total exacerbations and number of days of hospitalization due to exacerbations of COPD.

## Introduction

Chronic obstructive pulmonary disease (COPD) is a treatable, preventable, chronic lung disease that accounts for years lived with disability [1] and reduced life expectancy [2]. The prognosis of COPD depends on multiple factors [3]. From previous research, it is known that hospitalization for an acute exacerbation of COPD is associated with poor prognosis and increased risk of death [4]. A substantial proportion of patients dies within 1 year after being discharged from their first hospitalization for an exacerbation of COPD [5]. Patients with COPD Global Initiative for Chronic Obstructive Lung Disease (GOLD) [6] stages 3 or 4 (severe and very severe airway obstruction, respectively) have the highest risk for an exacerbation, although patients with COPD GOLD 2 (moderate airway obstruction) are also at risk [4]. The costs of COPD rise with increasing severity of exacerbations, with hospital admissions accounting for most of these costs [7]. Tools to prevent or shorten hospital admissions are necessary to slow down COPD progression and to limit health care costs.

eHealth interventions are promising for improving outcomes and decreasing costs in chronic diseases, including COPD [8,9]. Different types of eHealth interventions for COPD exist, ranging from apps to support self-management to telemonitoring programs in which patients are followed extensively [10]. Diverse outcomes in various settings with a variety of eHealth interventions have been studied. Previous studies have shown that eHealth could decrease exacerbations and hospital admissions in COPD patients [11,12]. Of those studies, one offered patients who were discharged from the hospital (admission due to an exacerbation of COPD) an intervention that included a comprehensive assessment at discharge, education, an individually tailored care plan, weekly phone calls, and access to a specialized nurse at the hospital through an online platform. The intervention resulted in a reduction in hospital admissions [12]. Another study included patients with COPD GOLD stages 3 and 4 who were seen by a pulmonologist. Patients were monitored via home-based telemonitoring that consisted of a device with a large screen and 4 buttons that patients used to fill out a daily questionnaire. Patients received feedback from their device, and the responses were also sent to a secure data center. The responses were categorized and prioritized, and respiratory nurses contacted the patients if values were alarming. After 6 months, there was a decrease in hospital admissions and exacerbations, and there was a tendency toward decreased number of days in the hospital and outpatient visits [13].

It is thought that patients with frequent exacerbations may benefit more from eHealth programs [14-18]. Despite the promising results from previous studies, no eHealth programs were incorporated in the latest COPD statement [6]. Based on previous research, for the current study, we hypothesized that giving patients the responsibility to act on signs of a COPD exacerbation and make them aware of changes in COPD symptoms and physical activity will influence self-management, which can lead to a reduction of exacerbations and hospitalizations. This was incorporated in the EmmaCOPD eHealth program. A new item in the intervention was the involvement of informal care givers (“buddies”). The Bravis Hospital (Roosendaal, The Netherlands) offered COPD patients who are at risk of exacerbations the possibility to use this EmmaCOPD program, consisting of an app that includes questionnaires and an activity coach. This program was designed to recognize signs of a COPD exacerbation and inform patients and buddies when symptoms worsened or the number of steps per day declined.The primary aim of this study was to determine the effect of EmmaCOPD on the number of days of hospitalization. The secondary outcome of this study was to assess the effect of this program on the number of total exacerbations.

## Methods

### Study Design

This was a retrospective study with a pre-post research design using real-world data that were retrieved from the electronic record system at the Bravis Hospital and from EmmaCOPD. Data were collected between April 2018 and December 2019 from patients who agreed to participate in EmmaCOPD. Analyses were performed between January 2020 and March 2020. Due to the retrospective nature of the study and the fact that this study does not fall under the Medical Research Involving Human Subjects Act (in Dutch, Wet medisch‐ wetenschappelijk onderzoek met mensen [WMO]), there was no need for ethical approval. Patients were aware that this intervention was new in clinical practice. All patients signed informed consent to use their data for research.

### Study Population

Patients could be included if they were treated by a pulmonologist in the Bravis Hospital. Patients were eligible for the intervention if they had COPD and if they had at least 2 exacerbations of COPD in the previous 12 months. An exacerbation was defined as an increase in symptoms that was more than day-to-day variation combined with prescription of a course of oral corticosteroids or antibiotics. Patients could also be included if they were at increased risk of exacerbations according to their health care provider.

Patients were excluded if they used EmmaCOPD before April 2018, because these patients could already have experienced the beneficial effects of the intervention. Furthermore, patients were excluded if they did not own an Android-based smartphone since the app for the Activity coach was only compatible with Android-based smartphones.

### EmmaCOPD Intervention

**Figure 1:**
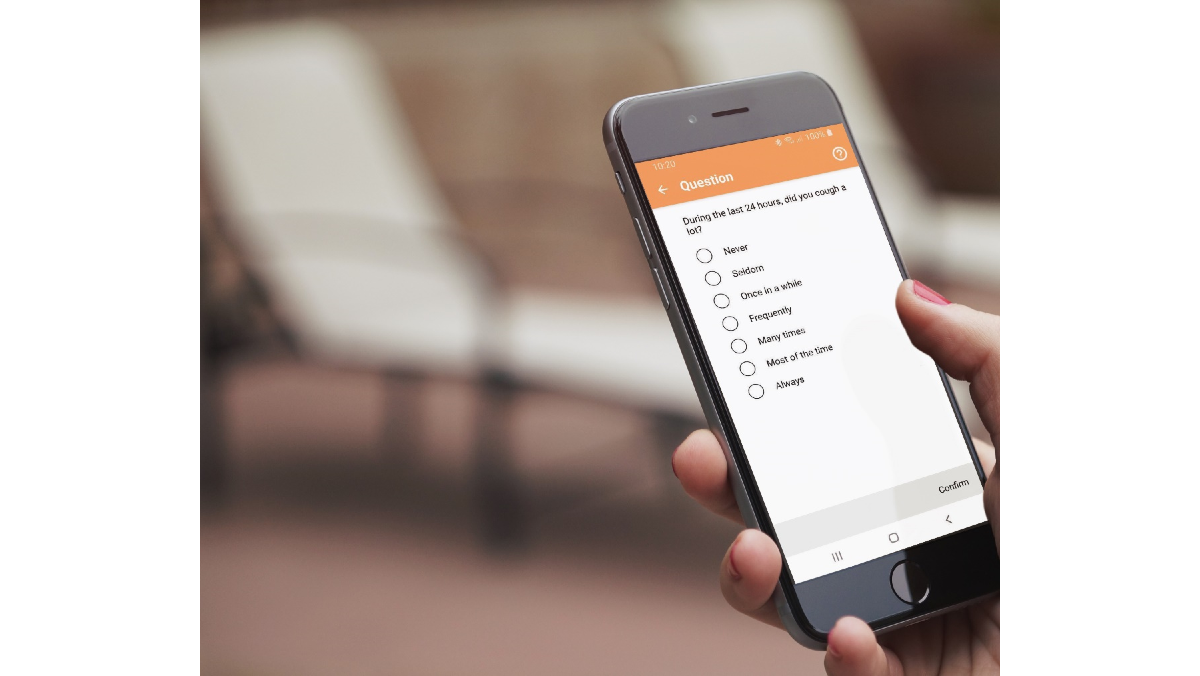
Questionnaire app to monitor symptoms.

**Figure 2:**
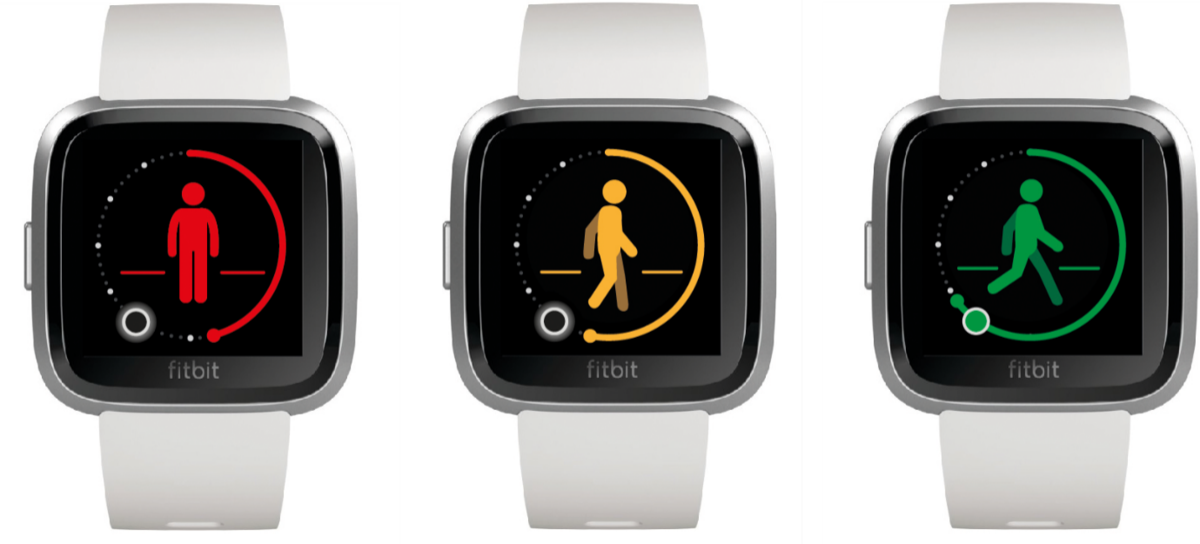
Smartwatch with built-in step counter.

**Figure 3:**
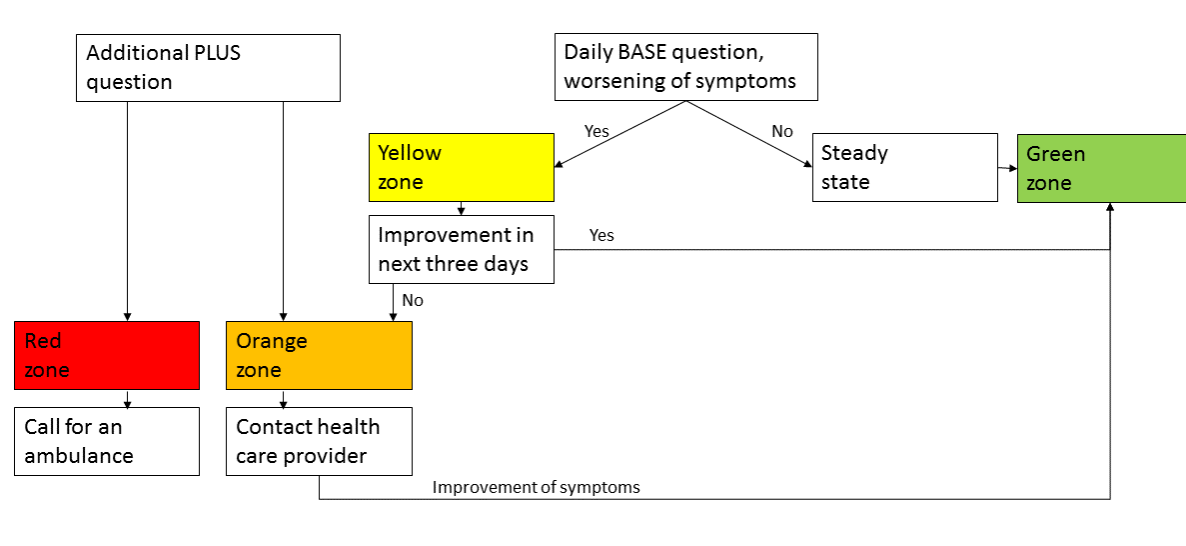
Flow chart with zones, based on questions.

Starting in November 2016, the Bravis Hospital (Roosendaal, The Netherlands) offered patients with COPD who are at risk for hospitalization due to an exacerbation a new eHealth program: EmmaCOPD, an app [19] that was designed to recognize signs of an exacerbation of COPD. EmmaCOPD was developed by Medicine Men BV (Utrecht, The Netherlands), with input from patients with COPD and physicians. The app used questionnaires (to monitor symptoms) and a step counter (to monitor the number of steps) to detect exacerbations (Figure 1 and Figure 2). Patients received feedback when their symptoms worsened or the number of steps declined. A “buddy” received this information too. A buddy was an informal caregiver who was close to the patient; this could be a relative, good friend, or neighbor. Health care professionals could be contacted by the patient or buddy if the app advised them to do so or if the patient or buddy was worried, but health care professionals were not involved in the fast response (Figure 3). Health care providers had access to the Emma account, and the account could be checked if it was needed.

In the program, patients could be in several zones: green, yellow, orange, or red. At the start of app use, patients were in the “green zone” (steady state). Every day, patients filled out the BASE questionnaire of the COPD action plan [20]. In the BASE questionnaire, patients reported whether they experienced worsening of COPD symptoms (Figure 3). If symptoms worsened (eg, shortness of breath; viscous sputum; sputum color changed to green, brown, or grey; wheezing; cough; fatigue; activities are difficult due to symptoms; headache; dizziness while awake or concentration problems), patients entered a so-called “yellow zone,” and they were advised to read their individual exacerbation plan and adapt their medication accordingly. If there was no improvement in 3 days, patients entered the “orange zone,” and the app advised them to either take the emergency medication or to contact a health care professional. When there was an improvement, the patient went back into the “green zone.” At any time, the buddy received a signal (SMS, email, app signal, dashboard signal, or a combination) when the status changed to another color. If the patient or buddy suspected something serious, they could fill out (at any time) a second question [20] (PLUS question, see Figure 3). In response to some answers on the PLUS question (eg, hemoptoe, fever or too sick to do activities), the patient entered the “orange zone” (see description earlier in the paragraph). For answers indicating a potentially life-threatening situation (eg, very dyspneic, chest pain, confused, forgetfulness, dizziness, tendency to collapse or loss of consciousness), the patients entered the “red zone,” and the buddy was advised to call for an ambulance immediately. Furthermore, patients completed the Clinical COPD Questionnaire (CCQ) [21] 3 times on a weekly basis to give insight into their COPD-related health status.

The Pebble Time, a modern programmable smartwatch that includes an accelerometer and gyrometer (Bosch Sensortec BMI160, BOSCH, Germany) was used to signal a decline in the number of steps. During the first 3 weeks, baseline activity level was assessed. Thereafter, a physiotherapist set a step goal. When patients reached this goal, patients were in the “green zone.” If there was a decline of 20% in the number of steps, patients received a signal that they entered the “orange zone”; if there was a decline of 40%, patients were in the “red zone.”

During an onboarding session in the Bravis Hospital, patients and buddies were prepared for the use of EmmaCOPD. Their individual exacerbation plan was checked, and an Emma account was created. Furthermore, the Emma questionnaire app and the Emma activity coaching companion app were installed on the patient’s smartphone. The buddy, health care providers, physiotherapists, and the support department of Medicine Men had access to the account.

### Data Collection

Baseline characteristics of participants were collected from the electronic health records at the Bravis Hospital. At baseline and follow-up, exacerbations were collected from the electronic records. At baseline, the number of exacerbations in the previous 12 months was collected. A mild exacerbation was defined as a “flare up of COPD symptoms with a change in COPD medication,” a moderate exacerbation as a “flare up of COPD symptoms that requires prescription of a course of corticosteroids or antibiotics,” and a severe exacerbation as a “flare up of COPD symptoms that led to hospital admission.” At baseline and follow-up, the number of hospitalizations and the number of days admitted to the hospital were collected. GOLD category (A, B, C, and D) at baseline was determined using the number of exacerbations and modified Medical Research Council score [6]. Data on how often patients filled out the questionnaires, results of the CCQ, and how often patients entered the orange and red zones were collected from EmmaCOPD.

### Power Calculation

From previous research, it is known that of all patients with COPD GOLD stage 2, 7% was admitted to the hospital. Of all patients with COPD GOLD stage 3, 18% was admitted. Of all patients with COPD GOLD 4, 33% was admitted [22]. Since the target population was treated by secondary care pulmonology, we estimated that 25% of patients were admitted to the hospital within 1 year. The mean number of days admitted to the Bravis Hospital due to exacerbation of COPD was 6.0 days. When exploring this number of admission days for all patients, the mean number of days admitted to the hospital within 1 year was 1.5 days per person per year. In 2014, one of the goals for the Long Alliantie Nederland was a 25% reduction in the number of days admitted due to exacerbation of COPD [23]. The new number of admission days was calculated to be 1.125 days per person per year. With an alpha of 0.05, power of 80%, mean of 1.5 days (SD 0.75 days) preintervention, mean of 1.125 days (SD 0.56 days) postintervention, correlation of 0.4, and drop-out rate of 20%, 40 patients were needed with a follow-up period of 1 year. We expected that we needed 8 months to include the patients.

### Statistical Analysis

Descriptive statistics are presented as mean (SD) for continuous variables with a normal distribution, median (IQR) for continuous variables without a normal distribution, and percentages for categorical variables. We used a pre-post research design. For the postintervention period, the follow-up duration was calculated as the number of days between the date of data extraction and the date of inclusion. If a patient died, the follow-up duration was calculated as the number of days between the date of death and date of inclusion. We compared the postintervention period with a preintervention period of 365 days before inclusion in the study. We analyzed the difference between the first CCQ score and the last CCQ score using descriptive statistics. Generalized estimating equations were used to analyze CCQ change over time. For the analysis of the effect of the intervention on the number of hospital admissions and the number of hospital admission days, which can be conceptualized as count data with repeated intra-individual measurements before and after initiation of EmmaCOPD, we used generalized estimating equation models. The distribution of the data was tested first to check whether the data fitted best with a Poisson distribution or negative binomial model. Outcomes are expressed as the incidence rate ratio (IRR). As explanatory variables, we used the length of follow-up (log-transformed) and intervention (coded as 0 or 1 for the preintervention and postintervention periods, respectively). Additionally, we adjusted for potential confounders of sex and age (model 2). In a final model, we additionally adjusted for baseline severity expressed as GOLD category (model 3) and inhaled corticosteroid use (model 4).

All analyses were conducted in SPSS version 25.0.

## Results

Baseline characteristics are presented in Table 1; 29 patients were included with a mean age of 67.4 years (SD 8.0 years), mean forced expiratory volume in 1 second % predicted of 45.5 (SD 17.7). In the 12 months before baseline, patients had a median of 2.0 (IQR 2.0-3.0) severe exacerbations and were admitted to the hospital for a median of 8.0 days (IQR 6.0-16.5 days).

**Table 1:** Baseline characteristics of patients with chronic obstructive pulmonary disease (COPD; n=29).

Patient characteristics		Results
Age (years), mean (SD)		67.4 (8.0)
Sex (women), n (%)		13 (45)
Ethnicity (Caucasian), n (%)		26 (90)
BMI (kg/m^2^), mean (SD)		27.3 (5.0)
Comorbidity (CCI^a^ score), mean (SD)		2.1 (1.2)
Asthma (yes), n (%)		3 (10)
**Smoking status, n (%)**		
	Current	3 (10)
	Ex-smoker	25 (86)
	Never	1 (3)
**Pulmonary medication, n (%)**		
	ICS^b^ mono (yes)	2 (7)
	LABA^c^ mono (yes)	6 (21)
	LAMA^d^ mono (yes)	11 (38)
	ICS/LABA in one device (yes)	14 (48)
	LABA/LAMA in one device (yes)	3 (10)
	Oral corticosteroids (yes)	13 (45)
Physical activity (steps a day), median (IQR)^e^		2482.5 (1394.3-4184.3)
**Pulmonary function, mean (SD)^e^**		
	FEV_1_^f^ (L)^e^	1.3 (0.6)
	FEV_1_ (% predicted)^e^	45.5 (17.7)
	FVC^g^ (L)^e^	2.9 (0.8)
	FVC (% predicted)^e^	82.0 (16.5)
	FEV_1_/FVC^e^	41.2 (14.3)
**Exacerbations**		
	Number of mild exacerbations^h^ in previous 12 months, median (IQR)	0.0 (0.0-0.0)
	Number of moderate exacerbations^i^ in previous 12 months, median (IQR)	0.0 (0.0-0.0)
	Number of severe exacerbations^j^ in previous 12 months, median (IQR)	2.0 (2.0-3.0)
	Total number of exacerbations in the previous 12 months, median (IQR)	2.0 (2.0-3.0)
	Number of patients with ≥2 exacerbations, n (%)	25 (86)
**COPD-related symptom scores, mean (SD)**		
	mMRC^k^ score^e^	3.0 (1.1)
	CCQ^l^ score^e^	3.0 (1.2)
**GOLD^m^-stage, n (%)^n^**		
	A^o^	0 (0)
	B^p^	1 (3)
	C^q^	1 (3)
	D^r^	21 (72)
Days admitted to the hospital due to COPD exacerbations, median (IQR)		8.0 (6.0-16.5)

^a^CCI: Charlson comorbidity index [24].

^b^ICS: inhaled corticosteroids.

^c^LABA: long-acting beta2 agonist.

^d^LAMA: long-acting muscarinic antagonist.

^e^n=26, data missing for 3 participants.

^f^FEV_1_: forced expiratory volume in 1 second.

^g^FVC: forced vital capacity.

^h^Mild exacerbation: change in COPD medication.

^i^Moderate exacerbation: course of corticosteroids and/or antibiotics.

^j^Severe exacerbation: hospital admission.

^k^mMRC: modified Medical Research Council.

^l^CCQ: clinical COPD questionnaire.

^m^GOLD: Global Initiative for Chronic Obstructive Lung Disease.

^n^n=23, data missing for 6 participants.

^o^A: low symptoms, low risk for exacerbation.

^p^B: high symptoms, low risk for exacerbation.

^q^C: low symptoms, high risk for exacerbation.

^r^D: high symptoms, high risk for exacerbation.

Outcomes at follow-up are found in Table 2. The median follow-up was 587.0 days (IQR 372-594 days), and 3 (3/29, 10%) patients died. A follow-up duration of at least 12 months was achieved by 23 of the 29 patients. The median numbers of mild, moderate, and severe exacerbations were 0.0 (IQR 0.0-0.0), 0.0 (IQR 0.0-0.0), and 1.0 (IQR 0.0-2.0), respectively. The median number of days admitted to the hospital was 2.0 days (IQR 0.0-4.0 days), with a maximum of 15.0 days. The median difference between the first and last CCQ scores was 0.3 points (IQR –0.4 to 0.9). The CCQ change over time was not statistically significant (*P*=.860).

The data for both the number of hospitalization days and total number of exacerbations fitted best within a Poisson distribution. Unadjusted analyses showed that, after initiation of the EmmaCOPD intervention, both the number of hospitalization days (IRR 0.210, 95% CI 0.116-0.382) and the total number of exacerbations (IRR 0.310, 95% CI 0.219-0.438) decreased significantly (Table 3). Analyses adjusted for age and sex showed comparable results, with a significant decrease in hospitalization days (IRR 0.209, 95% CI 0.114-0.382) and total number of exacerbations (IRR 0.310, 95% CI 0.217-0.435). Additional adjustment for GOLD category and inhaled corticosteroid use showed comparable results (Table 3).

**Table 2:** Follow-up at 12-18 months after initiation of EmmaCOPD (n=29).

Outcomes	Results
Follow-up duration (days), median (IQR)	587.0 (372.0-594.0)
Mortality (yes), n (%)	3 (10)
Number of mild exacerbations^a^, median (IQR)	0.0 (0.0 to 0.0)
Number of moderate exacerbations^b^, median (IQR)	0.0 (0.0 to 0.0)
Number of severe exacerbations^c^, median (IQR)	1.0 (0.0 to 2.0)
Total number of exacerbations, median (IQR)	1.0 (0.0 to 2.0)
Hospital admission (days), median (IQR)	2.0 (0.0 to 4.0)
Change in CCQ^d^, median (IQR)^e^	0.3 (–0.4 to 0.9)

^a^Mild exacerbation: change in COPD medication.

^b^Moderate exacerbation: course of corticosteroids and/or antibiotics.

^c^Severe exacerbation: hospital admission.

^d^CCQ: clinical chronic obstructive pulmonary disease questionnaire.

^e^n=28, data missing for 1 participant.

**Table 3:** Effect of EmmaCOPD on length of hospitalization and number of exacerbations, compared between 365 days before the initiation of EmmaCOPD and 12-18 months after the initiation of EmmaCOPD.

Analytic model	Hospitalization (days), IRR^a^ (95% CI)	Total number of exacerbations, IRR (95% CI)
Crude analysis (model 1)	0.210 (0.116-0.382)	0.310 (0.219-0.438)
Adjusted analysis (model 2)^b^	0.209 (0.114-0.382)	0.308 (0.217-0.435)
Adjusted analysis (model 3)^c^	0.225 (0.111-0.456)	0.327 (0.211-0.506)
Adjusted analysis (model 4)^d^	0.225 (0.111-0.456)	0.325 (0.208-0.508)

^a^IRR: incidence rate ratio.

^b^Adjusted for sex and age.

^c^Model adjusted for sex, age, and Global Iniative for Chronic Obstructive Lung Disease (GOLD) stage (patients with missing GOLD stage were exluded).

^d^Model adjusted for sex, age, GOLD stage (patients with missing GOLD stage were excluded), and inhaled corticsteroid use.

Data derived from EmmaCOPD are presented in Table 4. During the follow-up, the median number of daily BASE questions answered was 252.0 (IQR 125.0-423.0). The median number of answers on the BASE questions in the “yellow zone” (worsening of symptoms) was 26.0 (IQR 7.0-91.0), with a range of 0-527. Of the median 13.0 (IQR 5.0-68.0) PLUS questions answered, a median of 1.0 (IQR 0.0-4.0) was answered in the “orange zone” and 1.0 (IQR 0.0-3.0) in the “red zone.” The median numbers of days in the “orange zone” and “red zone” were 0.0 (IQR 0.0-25.0) and 3.0 (IQR 2.0-3.0), respectively. The median number of steps a day was 1710.0 (IQR 1144.0-3078.0).

**Table 4:** EmmaCOPD outcomes (n=29), with results categorized in zones (green, yellow, orange, or red), with each zone except green (steady state) requiring a different action.

Variables		Results
**BASE questions^a^**		
	Number of BASE question answered, median (IQR)	252.0 (125.0-453.0)
	Number of BASE questions answered as yes (yellow zone^b^), median (IQR)	26.0 (7.0-91.0)
**PLUS questions^c^**		
	Number of PLUS questions answered, median (IQR)	13.0 (5.0-68.0)
	Number of PLUS questions answered with an answer in the orange zone^d^, median (IQR)	1.0 (0.0-4.0)
	Number of PLUS questions answered with an answer in the red zone^e^, median (IQR)	1.0 (0.0-3.0)
**Zones**		
	Number of days in the orange zone^d^, median (IQR)	0.0 (0.0-25.0)
	Number of days in the red zone^e^, median (IQR)	3.0 (2.0-3.0)
Physical activity (steps per day), median (IQR)^f^		1710.0 (1144.0-3078.0)

^a^BASE question: daily question about worsening of symptoms;

^b^Yellow zone: patient experienced worsening of symptoms (BASE question yes) and given advice to adjust medication.

^c^PLUS question: additional question when patient or the patient’s buddy suspected something serious.

^d^Orange zone: no improvements in 3 days (yes answer to the BASE for 3 days) or an orange-rated answer to a PLUS question, for which the patient is given advice to take emergency medication or contact health care provider.

^e^Red zone: red answer on the PLUS question, potentially life-threatening clinical situation, buddy was advised to call an ambulance.

^f^n=27, data missing for 2 participants.

## Discussion

### Principal Findings and Comparison With Prior Work

The aim of the present study was to evaluate the effect of a new eHealth program (EmmaCOPD) on the number of hospitalized days and the total number of exacerbations in patients with COPD who are at risk for hospitalization. The present study, using real-world data, showed a significant decrease in the number of exacerbations and the number of days admitted to the hospital.

In line with the results of the present study, a Cochrane review [17] and a recent review [10] have shown that eHealth care programs and patient platforms were effective in reducing hospital admissions in COPD. Mostly, the effect of telemonitoring was studied. A previous study used home-based telemonitoring to monitor patients. The home-based telemonitoring consisted of a device with a large screen and 4 buttons that patients used to fill out a daily questionnaire. Patients received feedback from their device, and the responses were also sent to a secure data center. The responses were categorized and prioritized, and respiratory nurses contacted the patients if values were alarming. After 6 months, there was a decrease in hospital admissions and exacerbations, and there was a tendency toward decreased number of days in the hospitals and outpatient visits [13]. Another study examined an intervention for patients that were discharged from the hospital. The intervention included a comprehensive assessment, an educational session, an individually tailored care plan, weekly phone calls, and access to a specialized nurse at the hospital through a digital platform. The intervention resulted in a reduced number of hospital admissions [12] and an increase in BMI [11], but there was no difference in dyspnea, lung function, and quality of life [11]. Other studies also reported no effect of eHealth on dyspnea and quality of life. One study evaluated internet-based dyspnea self-management support that included education, skills training, and coaching and found improvement in arm endurance exercises, but no differences in dyspnea and quality of life [11]. In line with the mentioned studies, this study showed a decrease in the number of total exacerbations. Patients were also monitored via an app and smartwatch, and they received feedback. These are elements that were found to be missing in previous apps, as found in a previous review [25]. Furthermore, a new element in the present intervention was the involvement of buddies who received alerts when the clinical situation changed. This could have resulted in a decline in anxiety due to frequent checks and involvement of buddies. However, we did not assess anxiety in this study and cannot test this hypothesis. Another element of the intervention that could have resulted in the reduction of admission days included improvement of self-management (fast and adequate medication adaptation according to the individual exacerbation plan, feedback, and maintenance of physical activity).

In the Netherlands, several eHealth projects aiming to prevent hospital admissions have been initiated. One project is focusing on education of health care providers and patients for early detection of mild and moderate exacerbations to prevent severe exacerbations [26]. Another project is using the Assessment of Burden of COPD tool [27] that was filled out during each visit to the health care provider to give insight into the burden of COPD and to increase quality of life and the quality of perceived care. The (preliminary) results of these studies show a decrease in the number of days between exacerbation onset and recognition [26], between recognition and action [26], and between cognition and general practitioner visits [26] as well as improvement in quality of life and perceived quality of care [27].

### Strengths and Limitations

A strength of this study was the use of real-world data. EmmaCOPD was implemented as part of usual care in the Bravis Hospital, not as part of a study. Therefore, the risk of bias associated with participating in a study was minimized. Subgroup analysis in a systematic review has shown that telemonitoring is effective in patients with COPD, as are interventions that last more than 6 months [15]. In this study, the intervention was integrated into usual care, and there was no end date. The patients could have benefited from these characteristics. Another strength of the study was how self-management was organized. To minimize the risk of respiratory-related mortality that was reported in previous self-management interventions [28], patients were clearly instructed how to react when symptoms worsened, and there was a second question built in the app when patients or buddies suspected something serious. Furthermore, buddies received alerts when the clinical situation changed.

This study has limitations. First, there were fewer patients included in the study than were calculated in the power calculation. The number of patients included in the study could probably have been higher if the app had also been available for iPhones. Also, eHealth interventions often face implementation challenges, including costs, that might explain why the sample size was not met [29]. The number of patients that were eligible for EmmaCOPD but were not willing to use EmmaCOPD is unknown. A previous study showed that 15.9% of patients reject eHealth when it is offered [25]. Still, there was a significant difference in the number of days of hospitalizations. More patients with more follow-up data could have resulted in a more precise difference in the number of hospitalization days. A second limitation is selection bias; patients with the strongest motivation will accept such an intervention program, while those who are not motivated to improve their COPD condition will refuse the intervention. From pulmonary rehabilitation, it is known that the most frequently mentioned reason for refusal is lack of interest [30]. However, not all patients were motivated to fill out the questionnaire on a daily basis; during the median study period of 587.0 days, the BASE questions were answered on a median of 252.0 days. It could also be true that patients felt too sick to answer the questions. This could have resulted in an underestimation of the number of days in the yellow, orange, and red zones. Furthermore, it is known that interest in eHealth declines over time [31]. Third, the study design can be seen as a limitation since we used a pre-post research design, and there was no parallel control group. The data were collected from electronic health records, which possibly resulted in missing data. The total number of exacerbations is possibly higher, since not all mild or moderate exacerbations that were treated by the general practitioner were processed in the digital records of the Bravis Hospital. Furthermore, all respiratory-related hospitalizations were included, since pneumonia, dyspnea, and exacerbation frequently got mixed up. Finally, this study is not generalizable to all COPD patients since this study included just patients who were at risk for hospitalization due to exacerbation of COPD and this was a highly symptomatic group with a median 26.0 days with worsening symptoms, with one patient that was in the “yellow zone” nearly the whole study period (527 days). However, especially for the patient group that is at risk of hospitalizations [32], there is a need for intensive support to prevent future hospitalizations.

### Future Research

For future studies, we recommend a study with a longer follow-up since it is known that interest in eHealth often declines over time, with fewer responses on alerts [33]. In this study, we observed that patients did not fill out the daily questionnaire on a daily basis. However, as the goal of the intervention was to decrease exacerbations, it does not necessarily mean that the questionnaire should be completed every day. Nevertheless, the impact of usage on the effect of EmmaCOPD would be of interest. To strengthen the conclusions of this study, a case-control design can be considered to control for similar background. Furthermore, studies with a larger number of included patients are preferable, so small differences in outcomes can be detected as well.

### Conclusion

EmmaCOPD, an eHealth program that includes an app that signalled symptoms, a smartwatch with step counter, and provision of feedback to the patient and buddies, seems to reduce the number of total exacerbations and the number of days of hospitalization due to exacerbation of COPD in this real-world study. The effects of long-term use of EmmaCOPD should be studied further in future studies.

## Abbreviations

CCI: Charlson comorbidity index
CCQ: clinical COPD questionnaire
COPD: chronic obstructive pulmonary disease
GOLD: Global Initiative for Chronic Obstructive Lung Disease
IRR: incidence rate ratio
